# Mapping the Dynamic Network Interactions Underpinning Cognition: A cTBS-fMRI Study of the Flexible Adaptive Neural System for Semantics

**DOI:** 10.1093/cercor/bhw149

**Published:** 2016-07-25

**Authors:** JeYoung Jung, Matthew A. Lambon Ralph

**Affiliations:** Neuroscience and Aphasia Research Unit (NARU), School of Psychological Sciences, University of Manchester, Manchester, UK

**Keywords:** anterior temporal lobe, bilateral system, cTBS, fMRI, semantic representation

## Abstract

Higher cognitive function reflects the interaction of a network of multiple brain regions. Previous investigations have plotted out these networks using functional or structural connectivity approaches. While these map the topography of the regions involved, they do not explore the key aspect of this neuroscience principle—namely that the regions interact in a dynamic fashion. Here, we achieved this aim with respect to semantic memory. Although converging evidence implicates the anterior temporal lobes (ATLs), bilaterally, as a crucial component in semantic representation, the underlying neural interplay between the ATLs remains unclear. By combining continuous theta-burst stimulation (cTBS) with functional magnetic resonance imaging (fMRI), we perturbed the left ventrolateral ATL (vATL) and investigated acute changes in neural activity and effective connectivity of the semantic system. cTBS resulted in decreased activity at the target region and compensatory, increased activity at the contralateral vATL. In addition, there were task-specific increases in effective connectivity between the vATLs, reflecting an increased facilitatory intrinsic connectivity from the right to left vATL. Our results suggest that semantic representation is founded on a flexible, adaptive bilateral neural system and reveals an adaptive plasticity-based mechanism that might support functional recovery after unilateral damage in neurological patients.

## Introduction

Human higher cognitive function is not localized to single brain region but, rather, reflects the interaction of a network of multiple brain regions that act in concert to achieve flexible cognitive behaviors. Previous studies have demonstrated these brain networks using functional or structural connectivity approaches (Honey et al. 2009; van den Heuvel et al. 2009). While these studies provide the topology of brain networks, they pretermitted a key aspect of this neuroscience principle: how brain regions interact in a dynamic fashion to achieve cognitive function. Here, we explored this issue targeting a higher cognitive function, semantic memory, by employing a combination of transcranial magnetic stimulation (TMS) and fMRI.

Semantic memory refers to our collective knowledge about words, pictures, objects, people, emotions, etc. The neural basis of semantic memory reflects a large-scale network of distributed, interconnected brain regions (Patterson et al. 2007; Binder et al. 2009). Accumulating, convergent evidence indicates that among these brain areas, the ATLs act as a transmodal hub (Lambon Ralph et al. 1999; Bozeat et al. 2000; Chan et al. 2001; Coccia et al. 2004; Pobric et al. 2007; Lambon Ralph and Patterson 2008; Pobric et al. 2009, 2010a, 2010b, 2010c; Binney et al. 2010; Peelen and Caramazza 2012) which interacts with distributed modality-specific association regions to generate coherent, generalizable semantic representations (Lambon Ralph and Patterson 2008; Lambon Ralph, Sage, et al. 2010). While the key regions within the semantic network have been localized, we know very little about the functionally related interactions within this network and how these generate a relatively robust system capable of withstanding partial damage (e.g., as observed in patients with partial unilateral ATL damage) (Lambon Ralph, Cipolotti, et al. 2010; Schapiro et al. 2013). Therefore, by using a combination of TMS, fMRI, and connectivity analyses, this study investigated the flexible, adaptive nature of the bilateral neural system for semantic representation.

Repetitive transcranial magnetic stimulation (rTMS) over left or right ATL generates slowed rather than interrupted semantic performance in healthy participants (Pobric et al. 2007, 2009, 2010a, 2010b, 2010c). Likewise, patients with unilateral ATL damage (either left or right) exhibit mild semantic impairment on sensitive timed assessments but perform much better overall than those with bilateral temporal damage (Lambon Ralph, Cipolotti, et al. 2010; Bi et al. 2011; Lambon Ralph et al. 2012). These findings mirror seminal investigations of unilateral versus bilateral ATL resections in nonhuman primates and 1 human case (Brown and Schafer 1888; Klüver and Bucy 1939; Terzian and Ore 1955) in which unilateral resection generated only a transient multimodal associative agnosia, whereas bilateral resection led to devastated semantic performance. These findings imply that the bilateral ATL semantic system is configured to be a damage-resistant, robust system.

The neural mechanism, by which the ATLs interact with each other and other regions to underpin semantic function in the intact and partially damaged situation, remains unclear. Formal computational explorations of a bilaterally configured ATL semantic system suggest that dual representational hubs lead to an inherently greater robustness to unilateral than bilateral damage (even when total damage is held constant) and that this difference is magnified through long-term plasticity-related changes postdamage (as observed in unilateral patients) (Schapiro et al. 2013). Additional clues come from a study of patients with variable receptive language skills poststroke (Warren et al. 2009); overall, the patients showed a decrease in inter-ATL functional connectivity relative to healthy controls. More importantly, their comprehension performance was predicted by the degree of remaining ATL interconnectivity, such that those with preserved ATL connectivity exhibited the best language performance.

In the current study, we investigated the neural interactivity underpinning the semantic system through a combined TMS-fMRI methodology. This approach explores how focal perturbation of a target region affects neural activity and effective connectivity across the network of functionally connected regions. In the first experiment, we applied a virtual lesion delivered by cTBS in healthy participants to confirm that cTBS over the left vATL induces an inhibitory effect on semantic performance as 1 Hz rTMS has been shown to in previous studies (Pobric et al. 2007, 2009, 2010a, 2010b, 2010c). Having confirmed the effect of vATL cTBS on semantic cognition, we then combined cTBS with fMRI. This “perturb-and-measure” approach (Paus 2005) has been successfully implemented in the motor domain to demonstrate that rTMS both decreased neural activity at the stimulated site and induced increased task-related activity in the nonstimulated hemisphere (Lee et al. 2003; O'Shea et al. 2007). The researchers suggested that the upregulation in nonstimulated hemisphere reflected adaptive short-term compensatory plasticity that may underlie functional recovery after stroke. Accordingly, we expected that cTBS over the left vATL would suppress semantically related neural activity at the target region and induce upregulation in the homologous right vATL as well as, potentially, in other parts of the semantic network. To test whether the upregulation of the right vATL reflected adaptive short-term plasticity, we used dynamic causal modeling (DCM) of our fMRI data. We hypothesized that if the upregulation of the right vATL is linked with an acute compensatory reorganization, we should expect increased facilitatory input from the unlesioned to the lesioned hemisphere to improve task performance.

## Materials and Methods

### Participants

Thirty-five healthy individuals participated in this study, with 10 participants in the behavioral experiment (4 males, mean age, 22.8 ± 2.9 years, range from 20 to 28 years) and 25 in the fMRI experiment (7 males, mean age, 21.9 ± 3.7 years, range from 19 to 34 years). Data from 2 participants in the fMRI experiment were discarded because of excessive head movements (over a voxel). All participants were native English speakers and right handed (Oldfield 1971). Written informed consent was obtained from all participants. The experiment was approved by the local ethics committee.

### Behavioral Experimental Design and Procedure

All participants performed a synonym judgment task and a number judgment task as a control task. The synonym judgment task was adapted from the previous offline-rTMS and fMRI studies targeting ATL (Pobric et al. 2007, 2010a, 2010b, 2010c; Binney et al. 2010; Visser et al. 2012). The task required participants to select which of 2 words was more related in meaning to a probe word. In each trial, 3 words were presented on the screen, a probe (e.g., handy) on the top, the target word (e.g., functional), and the unrelated word (e.g., receptive) at the bottom. The 3 words in each trial were matched for imageability (*P* = 0.46), word frequency (*P* = 0.99), and word length (*P* = 0.62). The number judgment task was designed to match the synonym judgment task for general difficulty level, which was also adapted from previous studies. Participants were asked to select 3 digit numbers (e.g., 325 and 367) that was closer to the probe number (e.g., 358) in numerical value.

Participants received TMS stimulation at the left vATL or control site on different days (counterbalanced across participants). The experiment consisted of 2 sessions, the baseline (no-TMS) session and the after TMS session. The baseline session was conducted before or 1 h after the TMS session, and the order of sessions was counterbalanced across participants to control TMS and order effects. Each session contained both tasks, each of which had 60 trials. The trial started with 500 ms fixation, then the stimuli were presented until response or 3000 ms. The order of trials was randomized. E-prime software (Psychology Software Tools Inc., Pittsburgh, PA, USA) was used to display stimuli and to record responses.

### fMRI Experimental Design and Procedure

All participants had 2 separate sessions on different days (for vATL or control site stimulation). The fMRI scan had 2 runs and each run consisted of 15 blocks of synonym judgment task interleaved with 15 blocks of number judgment task. Between the task blocks, there were fixation blocks for 8 s. A task block was composed of 4 trials of each task. Each trial started with 1 s fixation followed by the stimuli presented for a fixed duration of 3 s. The total time of a run was 12 min. The run required the participants to press one of 2 buttons designating 2 choices in a trial. The tasks were as same as the behavioral experiment.

**Figure 1.**
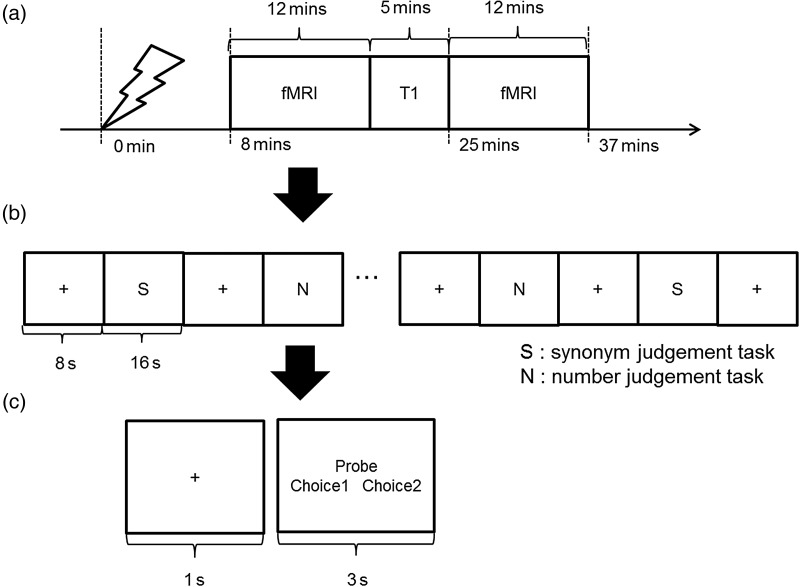
cTBS-fMRI experiment. (*a*) Procedure of an fMRI session. (*b*) Design of an fMRI block. (c) A schematic of the paradigm used in both synonym judgment and number judgment task.

Prior to each fMRI session, all participants received cTBS according to the stimulation sites outside of the scanner. Immediately after the stimulation, we transferred participants to the scanner, such that the first fMRI run started 8 min after the last cTBS pulse (Fig. 1).

### Transcranial Magnetic Stimulation

We applied cTBS (600 pulses at 50 Hz for 40s) at the stimulation sites with 80% of the individual resting motor threshold (RMT). RMT was defined as a minimal intensity of stimulation inducing motor-evoked potentials in the contralateral FDI muscle in at least 5 of 10 stimulation trials at the optimal scalp position. cTBS was conducted using a figure of 8 coil (70 mm diameter) connected to a MagStim Super Rapid stimulator (The MagStim Company, Whitland, UK). The average stimulation intensity (80% of RMT) was 49.4% ranging from 38% to 62% in the behavioral experiment and 49.1% ranging from 36% to 61% in the fMRI experiment.

Prior to the experiment, all participants were scanned to obtain a high-resolution *T*_1_-weighted anatomical image using a 3 T Philips MR Achieva scanner. The image had an in-plane resolution of 1 mm and a slice thickness of 1.8 mm with an acquisition matrix 256 × 256 voxels. To cover whole head, the number of slices was manipulated depending on head size.

**Figure 2.**
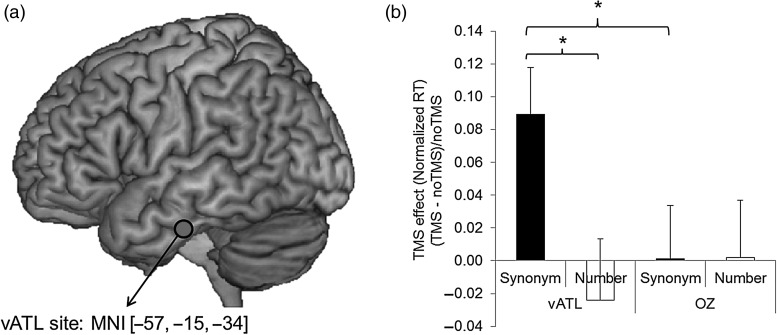
Behavioral experiment. (*a*) Left vATL TMS site. (*b*) Averaged TMS effects across participants for synonym judgment and number judgment tasks after cTBS over the left vATL or the control site (Oz). Error bar represents standard errors. *P< 0.05 (*P* values were corrected by FDR procedure).

Previous rTMS studies have used a lateral ATL stimulation site 10 mm posterior to the tip of temporal pole on the middle temporal gyrus (Pobric et al. 2007; Pobric et al. 2009, 2010a, 2010b, 2010c). This area was chosen to fall squarely within the broader ATL region that is commonly atrophied in semantic dementia patients (Binney et al. 2010). However, recent distortion-corrected fMRI studies (Binney et al. 2010; Visser et al. 2012) have shown that there are graded differences in the activation pattern across the ATL region with the maximal peak activation in the ventral ATL region. Thus, we selected the coordinate for the vATL from a previous distortion-corrected fMRI study (Visser et al. 2012) and the peak coordinate of the left vATL was [−57, −15, −24] (MNI; Montreal Neurological Institute). To make sure of targeting the vATL (located on the inferior temporal gyrus), we modified the coordinate to [−57, −15, −34] (Fig. 2*a*) and transformed this vATL coordinate to each participant's native space. This was achieved by normalizing each participant's MRI scan against the MNI template using Statistical Parametric Mapping software (SPM8, Wellcome Trust Centre for Neuroimaging, London, UK). Then, the inverse of each resulting transformation was used to convert the group MNI coordinate to the untransformed individual naïve space coordinate. These native-space vATL coordinates were used to guide the frameless stereotaxy, the Brainsight TMS-MRI co-registration system (Rogue Research, Montreal, Canada). The occipital pole (Oz) was used as a control site using international 10–20 system.

### fMRI Acquisition

All imaging was performed on a 3 T Philips Achieva scanner using an 8-element SENSE head coil with a SENSE factor 2.5. To compensate the signal dropout around rostral temporal cortices, we utilized a dual-echo fMRI protocol developed by Halai et al. (2014). The fMRI parameters included 42 slices, 96 × 96 matrix, 240 × 240 × 126 mm FOV, in-plane resolution 2.5 × 2.5, slice thickness 3 mm, and TR = 2.8 s. The 2 echo times were a short TE, 12 ms and a long TE, 35 ms. The total volume of 1 run was 258. A high-resolution *T*_1_-weighted structural image was acquired using a 3D MPRAGE pulse sequence with 200 slices, in-planed resolution 0.94 × 0.94 mm, slice thickness 0.9 mm, TR = 8.4 ms, TE = 3.9 ms.

### fMRI Analysis

First, the dual gradient echo images were extracted and combined using in-house MATLAB code (Halai et al. 2014). SPM8 was used for all preprocessing and the general linear model (GLM) analysis. Functional images were corrected for motion artifacts and different signal acquisition times by shifting the signal measured in each slice relative to the acquisition of the middle slice prior to combining the short and long echo images. The individual *T*_1_-weighted image was coregistered to the mean functional EPI image and segmented using the DARTEL (diffeomorphic anatomical registration through an exponentiated lie algebra) toolbox (Ashburner 2007). DARTEL provided gray and white matter templates that represent the brain size and shape of all participants and invertible and smooth deformations (flow fields) for each individual's native space image to this common coordinate space. Then, normalization was performed using DARTEL to wrap and reslice images into MNI space resampled to 1.5 × 1.5 × 1.5 mm voxel size. The functional images were normalized to this *T*_1_-weighted image and resampled to 3 × 3×3 mm voxel size. Smoothing was applied with an 8 mm full-width half-maximum Gaussian filter.

Statistical analyses were carried out using a GLM. The 2 runs were analyzed using a fixed-effects model consisting of each task condition (semantic judgment, number judgment, and fixation) and motion parameters as regressors. Contrast images were computed to assess differences in activations between the semantic task and the control task (semantic judgment > number judgment) for each participant. Multisubject analyses were conducted using a random-effect model. Statistical threshold was set at *P* < 0.001 at the voxel level and *P* < 0.05 at the cluster level with at least 100 contiguous voxels after family-wise error (FWE) correction.

### Region of Interest Analysis

Five regions of interest (ROIs) were defined on the basis of the group result of the contrast (semantic judgment > number judgment) in the control session (Oz stimulation). The ROIs corresponded to the ventrolateral prefrontal cortex (vlPFC, peak activation: −54, 12, 18), the vATL (peak activation: −33, −9, −39), the posterior middle temporal gyrus (pMTG, peak activation: −57, −39, 6), the premotor (PMC, peak activation: −48, −3, −39), and the supplementary motor area (SMA, peak activation: 0, 6, 57). The homologous areas in the right hemisphere were also defined as ROIs (vlPFC = 54, 12, 18; vATL = 33, −9, −39; pMTG = 57, −39, −6; PMC = 48, −3, 39). All were defined as a spherical ROI with a radius of 5 mm. The MarsBar toolbox was used to define and analyze ROIs.

### DCM Analysis

DCM is a method to estimate and make the inference of interaction in a predefined set of brain regions in different experimental contexts (Friston et al. 2003). In DCM, hemodynamic signals are modeled as changes in local neuronal activity which is mediated by experimental inputs. The ensuing neuronal interactions, distributed among brain regions, are translated into the observed blood oxygenation level-dependent (BOLD) signal. The strength and direction of regional connectivity are calculated by comparing the observed regional BOLD responses with the BOLD responses predicted by the underlying neuronal model. Three types of parameters are estimated: the intrinsic connections independent of experimental perturbations; the changes in the intrinsic couplings between regions induced by the experimental conditions (i.e., modulatory effect); and direct influences of the external input or stimuli on a given region (i.e., driving input).

The first step in the DCM analysis was to define 3 intrinsic models (summarized graphically in Supplementary Fig. 4). All 3 models comprised the same 4 seed regions but varied the pattern of interconnections. There were 2 vATL (bilateral) seed regions. These were defined at the individual level from the first eigenvariate of the fMRI signal at a liberal threshold of *P* < 0.01 (uncorrected) within a sphere of 5 mm radius derived from the effect of interest. All 3 intrinsic models included the same bilateral connectivity between the vATLs. Given the use of visual stimuli in our experiment, we assumed that the activity of the vATL seeds was driven by visual areas. Accordingly, like the vATL areas, left and right visual seed regions were defined from the group contrast (semantic judgment > number judgment) in the control stimulation session (peak activation: −24 −84 −6; 21 −93 −3). Based on these 4 seed regions, we set up 3 intrinsic models representing potential hypotheses about interregional connectivity between the visual and vATL areas (in all subsequent modulatory models, the first intrinsic model framework was found to be the best model—which is shown in Fig. 6*a*, the other intrinsic models are shown for completeness in Supplementary Fig. 4). In the second step of the DCM analysis, we crossed the 3 intrinsic framework models with 3 types of modulatory model (resulting in 9 DCM models)—designed to test 3 potential effects of stimulation. The analyses were repeated separately for vATL and control Oz stimulation according to task block (the semantic task results for vATL stimulation—the effect of core interest in this study—are summarized in Table 1 and Fig. 6*c*,*d*; and for the control site stimulation in Supplementary Table 2; the control task results are summarized in Supplementary Fig. 5 and Table 3). We established the modulatory models utilizing a similar approach to a previous study which employed DCM in an exploration of language function (Hartwigsen et al. 2013). Utilizing the full time-series of data, we then applied a Bayesian model selection (BMS) random-effects analysis to determine which of the 9 DCM models were the most likely given the observed fMRI data (Stephan et al. 2009). Finally, we extracted individual-specific estimates for parameters of interest in the winning model, including the driving input, the strength of intrinsic connections between the left vATL and the right vATL, and the impact of the modulations on the intrinsic connections by cTBS at the left vATL. These parameters were entered into Bonferroni-corrected 1-sample *t*-tests to assess differences from zero and paired *t*-tests to assess the modulatory influences on interregional connectivity.

**Table 1:** Parameter estimates of the winning model for the vATL stimulation

Parameter	Mean	SD	*t*	*P* value
Intrinsic connection				
Left vATL → right vATL	0.004	0.001	27.31	<0.001**
Right vATL → left vATL	0.024	0.018	6.25	<0.001**
Left VC → left vATL	0.001	0.004	2.14	0.044
Right VC → right vATL	−0.004	0.014	−1.46	0.157
Modulation by cTBS				
Left vATL → right vATL	0.019	0.284	3.13	<0.005**
Driving input				
cTBS over left vATL	−0.002	0.001	−10.98	<0.001**
Left visual input	0.001	0.001	3.78	<0.001**
Right visual input	0.002	0.001	5.70	<0.001**

Note: **P < 0.01, 2-tailed, Bonferroni-corrected.

## Results

### Behavioral Experiment

We first investigated the effects of cTBS on semantic processing by comparing participants' performance on the synonym judgment task and the control task (number judgment) with and without cTBS over the left vATL and the occipital pole (Oz). Reaction times (RT) were examined using a repeated-measures analysis of variance (ANOVA) with task (synonym judgment vs. number judgment), site (left vATL vs. Oz), and TMS (TMS vs. no TMS) as within-subjects factors. There was a significant main effect of task (*F*_1,9_= 32.37, *P* < 0.001). The other main effects and interactions did not reach the significance level (*F*s < 3.04, *P*s > 0.12). To detect a task-specific TMS effect, a 2 × 2 ANOVA was conducted for each task separately. For the synonym judgment task, there were no significant main effects of site (*F*_1,9_= 0.17, *P* = 0.69) and TMS (*F*_1,9_= 2.65, *P* = 0.19), but the interaction was significant (*F*_1,9_= 6.64, *P* < 0.05). Post hoc paired *t*-tests evaluating the interaction revealed that RT for synonym judgment slowed following the left vATL stimulation relative to the baseline (no TMS) [*t*_(9)_ = 2.94, *P* < 0.05]. Note that the slowing effect of cTBS over the left vATL was highly consistent across the participants. 9 out of 10 participants showed increased RT after cTBS of the left vATL. For the control task, there were no significant effects found (*F*s < 0.64, *P*s > 0.45). Even though a substantial main effect of task was found in the 3-way ANOVA, subsequent analyses clearly demonstrated the task-specific TMS effect in RT only after vATL stimulation. The error rate was also examined in a repeated-measures ANOVA with task, site, and TMS as within-subject factors. There was a significant main effect of task (*F*_1,9_= 17.84, *P* < 0.05), but the other main effects and interactions were not significant (*F*s < 4.36, *P*s > 0.07) (see Supplementary Fig. 1).

To evaluate the TMS effects found in RT, we computed the normalized TMS effect (the RT difference between the TMS condition and no TMS condition divided by the RT in the no TMS condition). Planned comparisons (paired *t*-tests) were carried out for each task with and without TMS at each site. We found that cTBS over the left vATL significantly increased RT during the synonym judgment task compared with the control task [*t*_(9)_ = 2.16, *P* < 0.05] and compared with the control site [*t*_(9)_ = 3.14, *P* < 0.05] (Fig. 2*b*). These behavioral cTBS findings replicated previous 1 Hz rTMS studies which targeted the ATL using a variety of semantic tasks (Pobric et al. 2007, 2009, 2010a, 2010b, 2010c), adding further evidence to the observation that the vATL is a critical region for semantic processing. Additionally, for the purposes of the current investigation, this first experiment confirmed the use of ATL cTBS for combination with fMRI in the second “perturb-and-measure” experiment.

### cTBS-fMRI Experiment

The behavioral results in the cTBS-fMRI experiment were similar to the behavioral experiment conducted with a separate participant cohort. The behavioral measurements (accuracy and RT) were examined using a repeated-measure ANOVA with task (synonym judgment vs. number judgment) and site (left vATL vs. Oz) as within-subject factors. It should be noted that there was no baseline (No TMS) for the cTBS-fMRI experiment. For accuracy, as expected, there was no significant TMS effect found (*F*s < 1.84, *P*s > 0.19). For RT, there was a significant main effect of task (*F*_1,21_ = 10.44, *P* < 0.005) and an interaction between the task and stimulation site (*F*_1,21_= 12.66, *P* < 0.005). Post hoc paired *t*-tests evaluating the interaction revealed that RT for synonym judgment slowed following the left vATL stimulation compared with the control stimulation [*t*_(22)_ = −3.47, *P* < 0.005] (see Supplementary Fig. 2).

**Figure 3.**
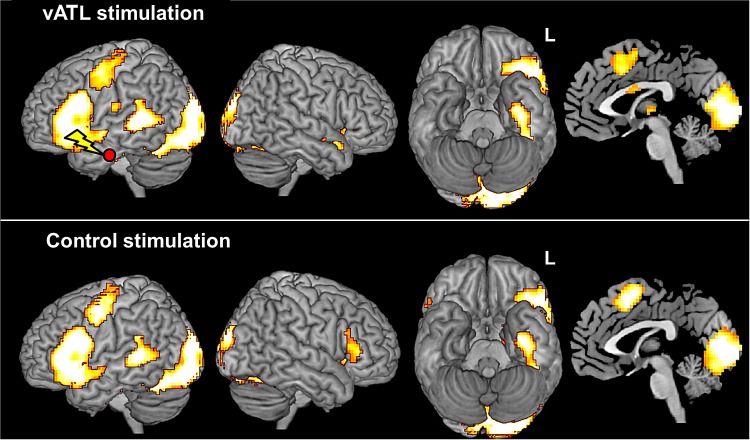
Brain activation maps of the synonym–number judgment contrast for the vATL stimulation (top) and the control stimulation (bottom). Activations displayed in the red/yellow color scale are survived at the cluster level, *P*_FWE-corrected_< 0.05 with at least 100 voxels.

To identify the neural network involved in semantic processing, we conducted a GLM analysis by contrasting synonym judgment with number judgment. A random-effects analysis after the control stimulation revealed strong activations in the left vlPFC (peak activation: −54, 12, 18; *z* = 6.82), the left vATL (peak activation: −33, −9, −39; *z* = 5.72), the left pMTG (peak activation: −57, −39, 6; *z* = 5.35), the left PMC (peak activation: −48, −3, 39; *z* = 5.73), the SMA (peak activation: 0, 6, 57; *z* = 5.38), and bilateral occipital cortex (peak activations: −12, −90, −9; *z* = 7.81 and 12, −87, −3; *z* = 7.72). Similarly, the activation map for the left vATL stimulation session showed strong semantic activations in the left vlPFC (peak activation: −57, 15, 21; *z* = 6.43), the left vATL (peak activation: −39, −15, −27; *z* = 5.25), the left pMTG (peak activation: −57, −36, 6; *z* = 6.58), the left PMC (peak activation: −45, −3, 51; *z* = 5.20), the SMA (peak activation: −3, 15, 54; *z* = 4.22), and bilateral occipital cortex (peak activations: −12, −87, −12; *z* = 7.76 and 18, −81, −6; *z* = 7.57) (Fig. 3; see Supplementary Table 1). This overall semantically related activation pattern replicated results from a recent distortion-corrected spin-echo fMRI study which employed in the same task (Binney et al. 2010). Furthermore, our data are consistent with previous studies that have shown vlPFC, vATL, and pMTG to be involved in semantic cognition (Binder et al. 2009, 2010; Visser et al. 2010, 2012; Noonan et al. 2013) plus PMC and SMA as reported in neuroimaging studies of reading and speech production (McGuire et al. 1996; Tremblay and Gracco 2006). The bilateral occipital activation was attributed to orthographic visual processing or semantic feedback to the visual areas (Hon et al. 2009). In short, the dual-echo fMRI task data confirmed that the typical full set of semantically related regions was activated by the task, and thus, this paradigm was a suitable one to explore the effects of vATL stimulation across this semantic neural network.

**Figure 4.**
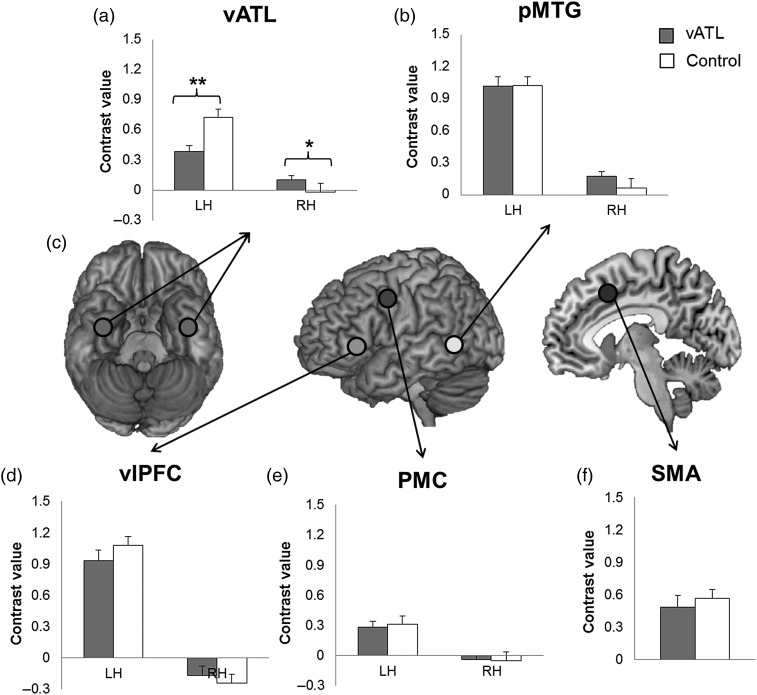
Effects of cTBS of the synonym–number judgment contrast for the vATL stimulation (gray color bar) and the control stimulation (white color bar) in ROIs (*a,b*,*d–f*). (*c*) Locations of ROIs. Error bar represents standard errors. *P< 0.05, **P< 0.01 (*P* values were corrected by FDR procedure).

Next, we examined the effect of cTBS over the left vATL. Five ROIs were determined by the results of the control stimulation (vlPFC, vATL, pMTG, PMC, and SMA). To explore the remote effect of cTBS during semantic processing, we also added the homologous areas in the right hemisphere (vlPFC, vATL, pMTG, and PMC). We observed that cTBS over the left vATL not only decreased activation at the targeted left vATL [*t*_(22)_ = 3.79, *P* < 0.01] but also increased activation at the homologous right vATL [*t*_(22)_ = −2.32, *P*= 0.05] (Fig. 4*a*). The right pMTG showed increased activation after cTBS at the left vATL compared with the control site [*t*_(22)_ = −2.06, *P* = 0.06] (Fig. 4*b*). The other ROIs did not show any significant changes (Fig. 4*d*–*f*). This analysis clearly demonstrated that cTBS successfully modulated activation at the target site and connected homolog regions. These finding confirmed our prediction that cTBS over the left vATL would alter regional activity in brain areas involved in semantic processing.

In addition, we investigated the effects of cTBS across the whole brain according to the stimulation sites by comparing the whole-brain activation maps of the left vATL with that of the control site. Relative to cTBS over the control site, cTBS over the left vATL significantly reduced deactivations in the posterior cingulate cortex (PCC, peak activation: 6, −36, 39; *z* = 4.20) and precuneus (peak activation: 12, −54, 33; *z* = 4.50) during synonym judgment compared with the control condition. We also found activity changes in the right vATL (peak activation: 51, 0, −33; *z* = 3.92) and bilateral angular gyrus (peak activation: −48, −63, 42; *z* = 3.31 and 42, −69, 39; *z* = 3.64) with a more liberal *P*_uncorrected_< 0.001 (10 voxels) after applying a small volume correction (SVC within a 10 mm sphere, *P*_FWE-corrected_< 0.05) in cTBS over the left vATL compared with the control site (see Supplementary Fig. 3).

**Figure 5.**
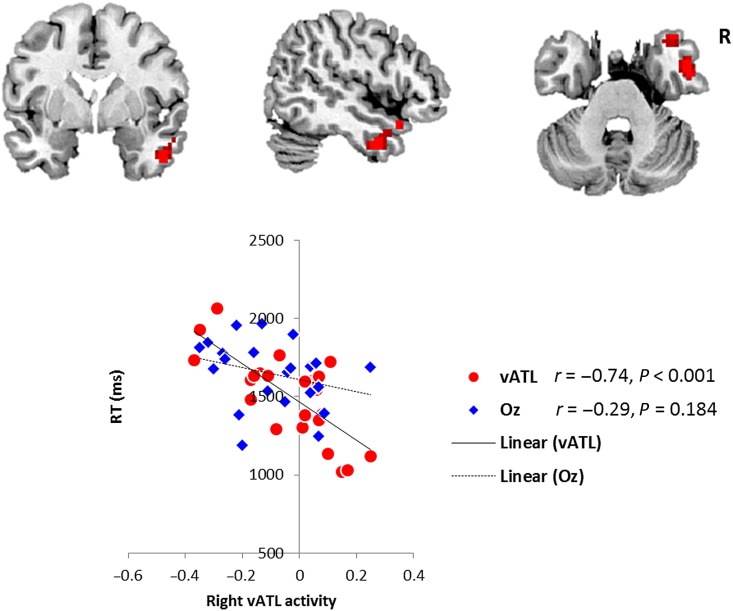
Right vATL activity predicted semantic RT after cTBS over the left vATL (top). Scatter plot shows the behavioral correlation in the right vATL following cTBS to either the left vATL or the control (Oz) site (bottom). Red circles represent semantically related individual activity in the right vATL after left vATL stimulation. Blue diamonds represent the same right vATL activation-behavioral correlations (nonsignificant) after control stimulation.

To explore the link between neural changes and task performance, we analyzed which region demonstrated a changed level of correlation with semantic RT performance after left vATL versus Oz stimulation. The right vATL was identified as the only significant region (peak activation: 51 0 −33, *P*_SVC-FWE_< 0.05). Following Oz stimulation, there was no significant correlation between the (semantic–control task) BOLD effect and task performance in this region. Yet following left vATL stimulation, there was a significantly enhanced relationship such that the right vATL's activity was negatively correlated with semantic task RT—that is, those participants with enhanced semantic performance (shorter RTs) had higher right ATL activation (Fig. 5).

### The Effect of cTBS on Effective Connectivity

**Figure 6.**
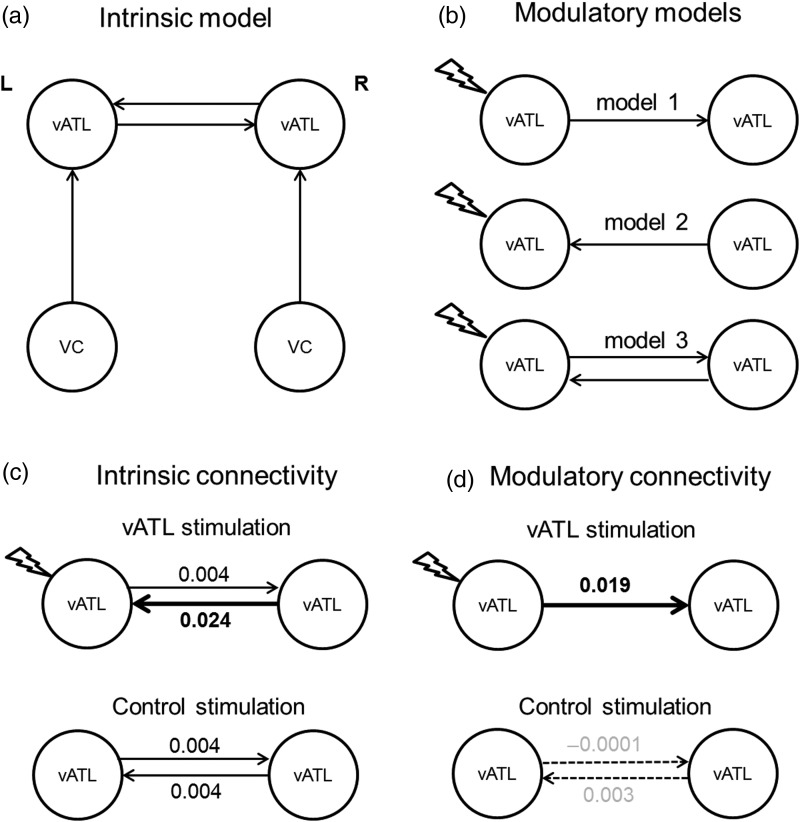
Results of semantic task DCM analyses (see Supplementary Fig. 5 for results of control task). (*a*) The intrinsic model with intrinsic connections (solid arrows) of bilateral vATL and from VC to vATL. (*b*) The 3 different modulatory motels tested. The models have the external modulation by cTBS over the left vATL with different set of modulatory connections (solid arrows). (*c*) The results of intrinsic connectivity. cTBS over the left vATL increased intrinsic connectivity from the right vATL to the left vATL compared with the control stimulation (fat solid arrow). (*d*) The results of modulatory connectivity. cTBS over the left vATL increased the semantically related modulatory connectivity of the winning model (fat solid arrow) compared with its intrinsic connectivity and control task connectivity. The modulatory connections of the control stimulation are not significant (dotted arrows).

We used DCM to explore the changes in the interactions between the vATL bilateral network after suppression of the left vATL by cTBS. If the neural structure of semantic processing is a bilateral system, as previously proposed (Lambon Ralph et al. 2001; Pobric et al. 2009; Schapiro et al. 2013; Lambon Ralph 2014), we would expect there to be increased facilitatory input from the right to left vATL after cTBS. To test this hypothesis, we defined the bilateral vATL as well as bilateral visual cortex (VC) as seeds, because semantic processing was triggered by visual stimuli in our experiment. We specified mutual intrinsic connections between vATL seeds and connections from VC to vATL in each hemisphere (Fig. 6*a*). Intrinsic models reflecting other possible visual input interactions were also established and tested (see Supplementary Fig. 4*a*). On the basis of this intrinsic model, we established 3 modulatory models expressing potential interregional connections between vATL seeds during semantic processing (Fig. 6*b*). Among 3 modulatory models tested, BMS (Stephan et al. 2009) confirmed model 1 as the winning model with a modulatory connection from the left vATL to the right vATL after cTBS of the left vATL for the semantic and control task (see Supplementary Fig. 4*b* top). For the control stimulation, same modulatory models without the driving (stimulation) input at the left vATL were tested, and model 3 was identified as the winning model (see Supplementary Fig. 4*b* bottom).

Our data demonstrated that cTBS over the left vATL significantly increased intrinsic connectivity from the right vATL to the left vATL [*t*_(22)_ = 6.25, *P* < 0.001] relative to the control stimulation (Fig. 6*c*) and control task [*t*_(22)_ = 5.07, *P* < 0.001] (see Supplementary Fig. 5). This finding supports our hypothesis that cTBS of the left vATL inhibited its activation and induced an adaptive upregulation of the homologous right vATL which, in turn, changed the intrinsic interregional connection to compensate current interruption in the system. The intrinsic connectivity after the control stimulation were facilitatory connections bilaterally [the left vATL → the right vATL: *t*_(22)_ = 59.88, *P* < 0.001; the right vATL → the left vATL: *t*_(22)_ = 37.01, *P* < 0.001]. It is important to note that, even in the “intact” system, the vATLs in the each hemisphere were mutually positively connected. Following cTBS over the left vATL, this semantically related bilateral connectivity was upregulated 5-fold such that it was significantly higher than the intrinsic connection [*t*_(22)_ = −3.07, *P* < 0.005] and also greater than the bilateral vATL functional connection following stimulation of the control region [*t*_(22)_ = 3.12, *P* < 0.005] (Fig. 6*d*). Furthermore, the modulatory connection of the semantic task was significantly stronger than that of the control task after cTBS over the left vATL [*t*_(22)_ = 3.14, *P* < 0.005] (see Supplementary Fig. 5). For the control task, the connectivity was not significant regardless of the stimulation site (*t*s < 1.64, *P*s > 0.12) (see Supplementary Fig. 5). It is noted that the modulatory connection after the control stimulation was not significant (*t*s < 1.17, *P*s > 0.26) (Fig. 6*d*). The details of this DCM analysis are summarized in Table 1 and Supplementary Tables 2 and 3.

## Discussion

Converging evidence has implicated the ATLs, bilaterally*,* as a crucial component in semantic representation. To date, however, it has been unclear how the 2 ATL regions interact to form a coherent functional system and in a manner that makes the system relatively robust to unilateral perturbation or damage. We were able to provide novel insights about these critical issues by disturbing the function of the left vATL with cTBS and using dual-echo fMRI to assess the consequences on behavior, changes in the level of activity, and alterations of interregional activity as well as interregional connectivity across the semantic network. Our results suggest that semantic representation is founded on a flexible, adaptive bilateral neural system and reveals an intrinsic adaptive plasticity-based mechanism that might support functional recovery after unilateral damage in neurological patients.

We observed that compared with cTBS over the control site, stimulation of the left vATL resulted in a selective slowing of semantic processing (mimicking the effect observed in patients after unilateral ATL resection) (Wilkins and Moscovitch 1978; Lambon Ralph et al. 2012). This inhibitory behavioral effect of vATL cTBS on semantic performance has been reported by previous studies which employed 1 Hz ATL rTMS to demonstrate transient selective semantic impairment across verbal and nonverbal domains as well as in receptive and expressive tasks (Pobric et al. 2007, 2009, 2010a, 2010b, 2010c). Importantly, for the purposes of the present study, it confirmed that we could use vATL cTBS to induce a “virtual lesion” that selectively affects semantic cognition.

The fMRI results showed that cTBS over the left vATL decreased the regional activity at the target site as well as increased activity at the homolog right vATL. This increased activity at the right vATL strongly predicted semantic performance only after left vATL stimulation, whereas there was no correlation following Oz stimulation. This finding supports our hypothesis that when the left vATL was perturbed by TMS stimulation, the homolog right vATL was upregulated to compensate the temporary disturbance in the semantic system. In addition, we observed that vATL cTBS evoked reduced deactivation in PCC/precuneus and angular gyrus, which are key regions in the default mode network (DMN). Previous investigations, which have differentiated the DMN from the semantic network, have demonstrated that both precuneus and AG “deactivate” in comparison with “rest” for a range of semantic and nonsemantic tasks, whereas ATL is positively activated for the semantic task (Lambon Ralph 2014; Humphreys et al. 2015). Thus, the reduced deactivation in the PCC/precuneus and AG after vATL stimulation might be related to a change in the recruitment of the DMN as general mental resource rather than semantic function.

To our best knowledge, this is the first study to demonstrate effective connectivity between left and right anterior temporal regions after a focal virtual lesion. The benefit of employing effective connectivity analysis is that it provides directional information about the interaction between areas. Previous studies have provided contrastive hypotheses about the role of the unlesioned homotopic region in language recovery following unilateral damage, which could be either beneficial or nonbeneficial (Chollet et al. 1991; Price and Crinion 2005; Hamilton et al. 2011). For example, we found that cTBS over the left vATL caused an upregulation in the right vATL. This upregulation could be interpreted in terms of an interhemispheric disinhibition of the right vATL, which results in a maladaptive overactivation. Alternatively, the right vATL might increase its (positive) contribution to sustain function after damage in the left vATL, leading to an adaptive upregulation. The DCM analyses showed that the increased activation in the right vATL, after the suppression of the left vATL, leads to enhanced connectivity from the right vATL to the left vATL. Accordingly, the results cannot be explained by a consequence of reduced interhemispheric inhibition caused by cTBS. If that were the case, we would observe a negative connectivity between vATLs without stimulation and a reduced negative connectivity from the left to right vATL after the left vATL stimulation. Rather, our findings support the alternative hypothesis by demonstrating that the perturbation on the left vATL produced an increased facilitatory drive from the right to the left vATL.

DCM analysis also demonstrated that, during the synonym judgment task, cTBS increased the facilitatory influence (modulatory connectivity) which may contribute to the upregulation in the right vATL. It would appear, therefore, that the cTBS-induced changes in modulatory connectivity reflect alterations to the existing intrinsic connectivity. Following stimulation of the control site, there was no significant task-related connectivity between vATLs, and thus, it would seem that cTBS modulates intrinsic connectivity which, in turn, influences the task-specific interregional connectivity (Grefkes et al. 2010).

Despite its potential power to reveal the network dynamics underpinning higher cognition, there are currently very few neuroimaging studies that have examined TMS-induced changes in language networks. One study, targeting Wernicke's area with rTMS, showed increased task-related activity in homologous brain regions contralateral to the stimulated sites during word recognition task (Andoh and Paus 2011). The researchers suggested that the increased activity in homologous areas after rTMS was a form of adaptive plasticity in the nondominant hemisphere reflecting an interhemispheric compensatory mechanism. Another study, which used cTBS to stimulate Broca's area, also showed an increase in the homologous brain region activation (Hartwigsen et al. 2013). Subsequent DCM analysis revealed an increase in the facilitatory drive from the right inferior frontal gyrus (IFG, nonstimulated region) to the left IFG (stimulated region) during speech processing, indicating another example of short-term plasticity. A recent rTMS study (Binney and Lambon Ralph 2015) also found evidence of right homolog upregulation of activity in response to left lateral ATL rTMS, although this study did not examine changes in the functional connectivity. Consistent with similar explorations of nonlanguage domains (Lee et al. 2003; Sack et al. 2005; O'Shea et al. 2007), these findings suggest that after transient inhibitory stimulation, a compensatory short-term reorganization can occur, with a positive upregulation of the contribution from the homologous area in the contralateral hemisphere. As well as providing important insights about the dynamic, rapid plasticity within intact neural networks, these results also suggest that this compensatory reorganization, driven from the homologous hemisphere, might be able to preserve and restore function—which is particularly important for considering the neural bases for long-term recovery of function after brain damage or neurosurgery.

A second linked feature of our results is the observation that the compensatory right vATL contribution to semantic function arose from the system being inherently bilateral in nature. Specifically, the DCM results indicated that there are positive, facilitatory, intrinsic interhemispheric connections between vATL regions. This result not only indicates that the adaptive change (following left vATL stimulation) is founded on the intrinsic connections, but it also fits with other data indicating that the semantic system is supported through a bilateral, interconnected ATL system (Pobric et al. 2009, 2010a, 2010b, 2010c; Lambon Ralph, Sage, et al. 2010; Lambon Ralph et al. 2012; Schapiro et al. 2013). Following classical studies of unilateral versus bilateral ATL resection in nonhuman primates and human participants (Brown and Schafer 1888; Klüver and Bucy 1939; Terzian and Ore 1955), recent computational models and neuropsychological data indicate that substantial semantic impairment only follows when ATL damage is bilateral in nature (Lambon Ralph et al. 2001; Patterson et al. 2007; Schapiro et al. 2013). In comparison, patients with unilateral damage (at least in the chronic phase after spontaneous recovery) can present with very much better, albeit not entirely normal, semantic function (Lambon Ralph, Cipolotti, et al. 2010; Bi et al. 2011; Lambon Ralph et al. 2012). The current cTBS-fMRI investigation might provide important clues about the mechanism which allows patients with unilateral ATL damage to function so well after a period of recovery. Specifically, the inherent bilateral interactions and rapid contralateral compensatory upregulation would allow the system, over time, to recovery semantic function. This hypothesis is consistent with a recent investigation of the functional connectivity of ATLs in chronic poststroke aphasic patients (Warren et al. 2009), which found that the patients' remaining degree of functional connectivity between (intact) anterior temporal regions lobes was correlated with their recovered comprehension performance. Taken together, these findings suggest that both of the left and right ATL play critical roles in semantic processing, not only individually, but also by interacting with each other through the white matter connections in the anterior commissure tracts (Demeter et al. 1990; Catani et al. 2002; Schmahmann and Pandya 2006).

## Supplementary Material

Supplementary material can be found at: http://www.cercor.oxfordjournals.org/online.

## Funding

The research was supported by a Medical Research Council (UK) MRC Programme Grant (MR/J004146/1) to M.A.L.R. Funding to pay the Open Access publication charges for this article was provided by a UK Research Councils (RCUK) award to the University of Manchester.

## Supplementary Material

Supplementary Data
