# Supplementary material for: Mapping the Dynamic Network Interactions Underpinning Cognition: A cTBS-fMRI Study of the Flexible Adaptive Neural System for Semantics
Source: Cereb Cortex. 2016 Jul 25;26(8):3580–90. doi: 10.1093/cercor/bhw149 (PMC4961025; doi:10.1093/cercor/bhw149)
Supplement: Supplementary Data [file supp_26_8_3580__index.html]

Mapping the Dynamic Network Interactions Underpinning Cognition: A cTBS-fMRI Study of the Flexible Adaptive Neural System for Semantics — Mapping the Dynamic Network Interactions Underpinning Cognition: A cTBS-fMRI Study of the Flexible Adaptive Neural System for Semantics — Supplementary Data 

# Mapping the Dynamic Network Interactions Underpinning Cognition: A cTBS-fMRI Study of the Flexible Adaptive Neural System for Semantics

## Supplementary Data

Supplementary Data

- Supplementary Data - Docx file
