# Supplementary material for: Mapping the Dynamic Network Interactions Underpinning Cognition: A cTBS-fMRI Study of the Flexible Adaptive Neural System for Semantics
Source: Cereb Cortex. 2016 Jul 25;26(8):3580–90. doi: 10.1093/cercor/bhw149 (PMC4961025; doi:10.1093/cercor/bhw149)
Supplement: Supplementary Data [file supp_bhw149_bhw149supp.docx]

**Mapping the dynamic network interactions underpinning cognition: a cTBS-fMRI study of the flexible adaptive neural system for semantics**

JeYoung JUNG & Matthew A. LAMBON RALPH

**Supplementary Fig.1
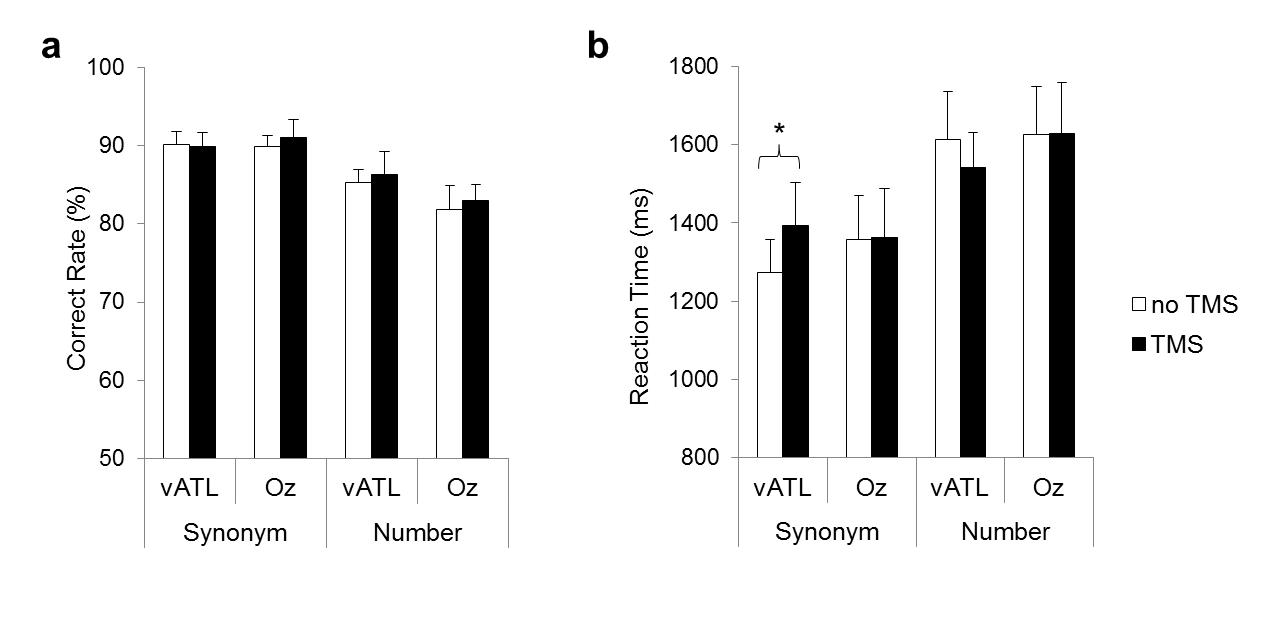
**

Supplementary Figure 1. Averaged performance of synonym and number judgment task. (a) Accuracy (B) Reaction time. Error bar represents standard errors. * *P* < 0.05 (P values were corrected by FDR procedure).

**Supplementary Fig. 2**

**
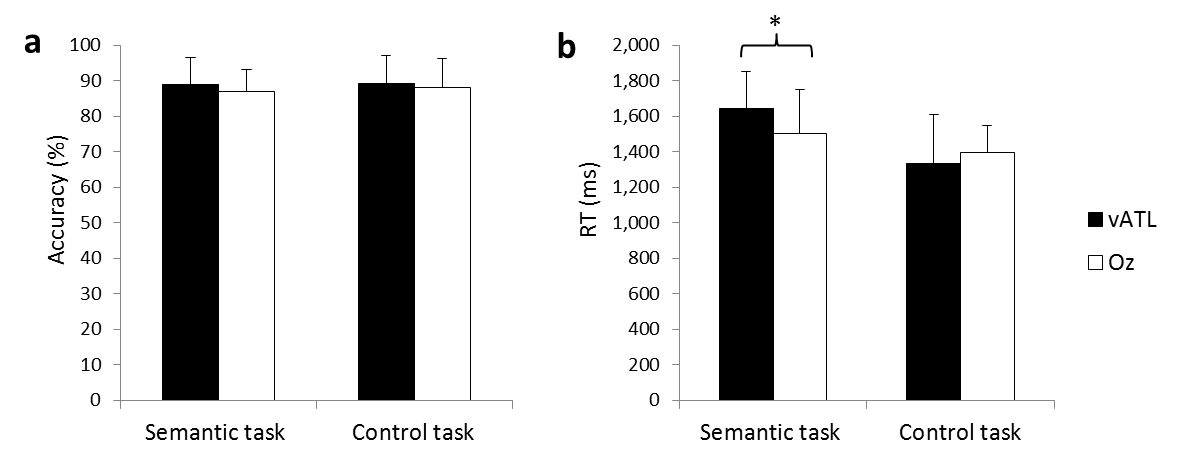
**

Supplementary Figure 2. The behavioural results of cTBS-fMRI experiment. (a) Accuracy (b) Reaction time. Error bar represents standard errors. * *P* < 0.05. Black bar represents the vATL stimulation. White bar represents the control stimulation.

**Supplementary Fig. 3**

**
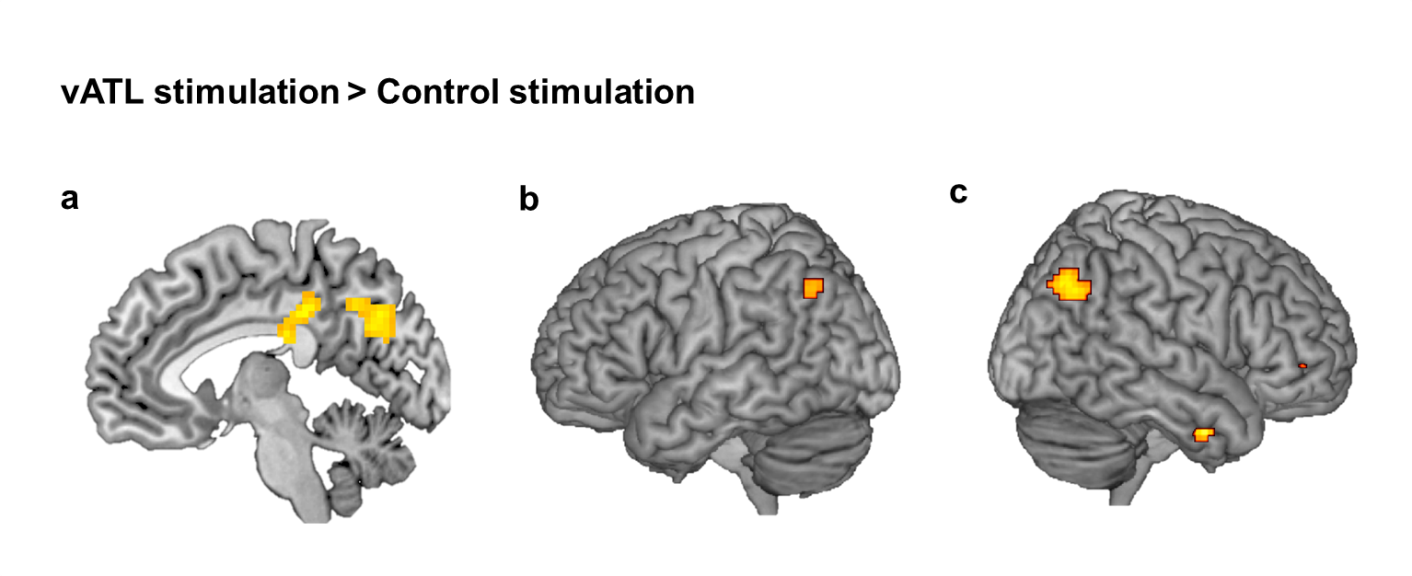
**

Supplementary Figure 3. Brain activation maps of vATL stimulation > control stimulation during synonym judgement. (a) Differential deactivation in precuneus and posterior cingulate cortex, which survived at the cluster level, *P* _FWE-corrected_ < 0.05 with at least 100 voxels. (b) Differential deactivation in the left angular gyrus. (c) Differential deactivation in the right angular gyrus and the right vATL. **Supplementary Figure 4**

**
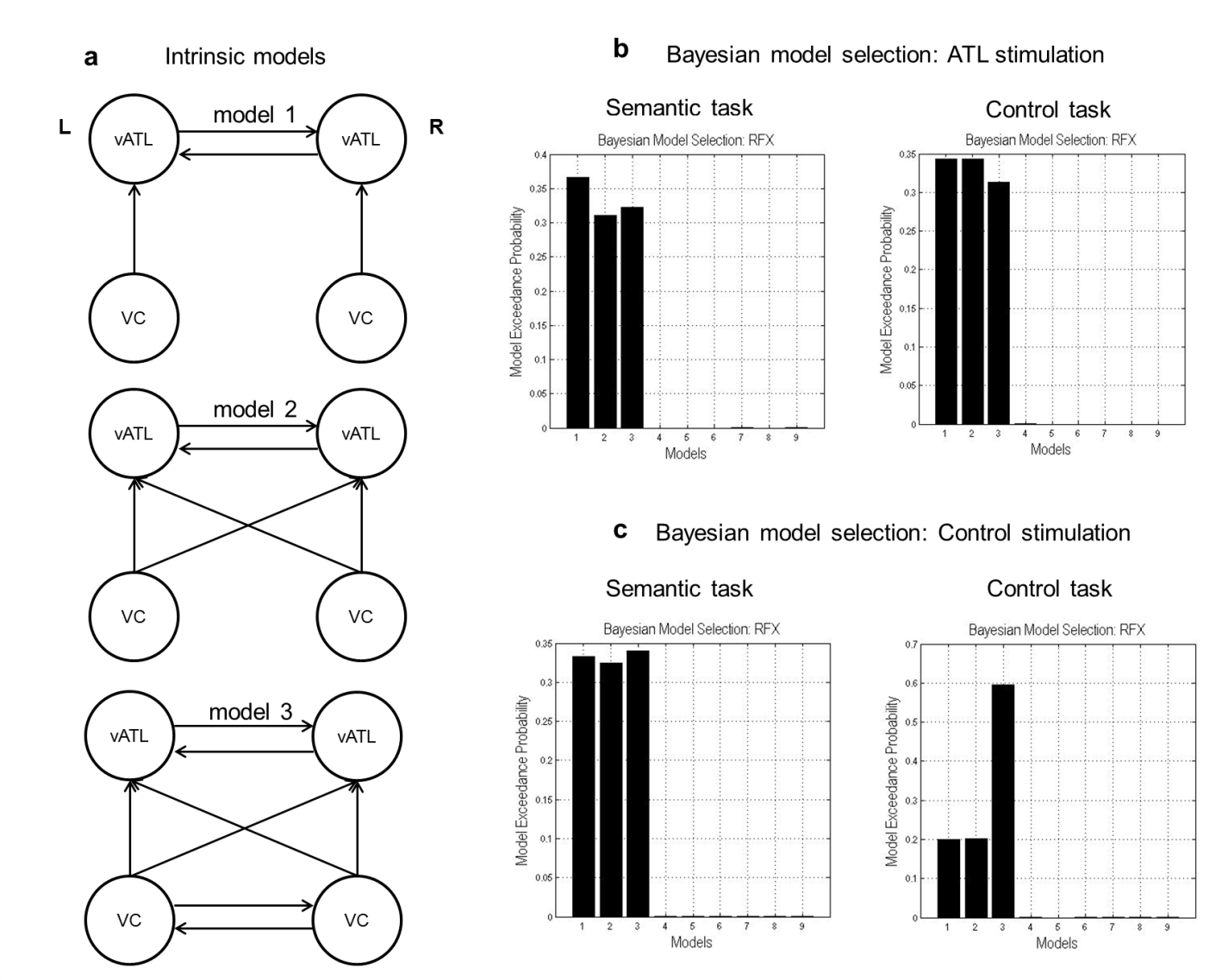
**

Supplementary Figure 4. (a) Intrinsic models. (b) Bayesian model selection: ATL stimulation Based on the three intrinsic models, nine modulatory models were established and tested for each task separately. (c) Bayesian model selection: Control stimulation. Model 1, 4, 7 were modulatory model 1. Model 2, 5, 8 were modulatory model 2. Model 3, 6, 9 were modulatory model 3 (See Fig. 6b).

**Supplementary Figure 5**

**
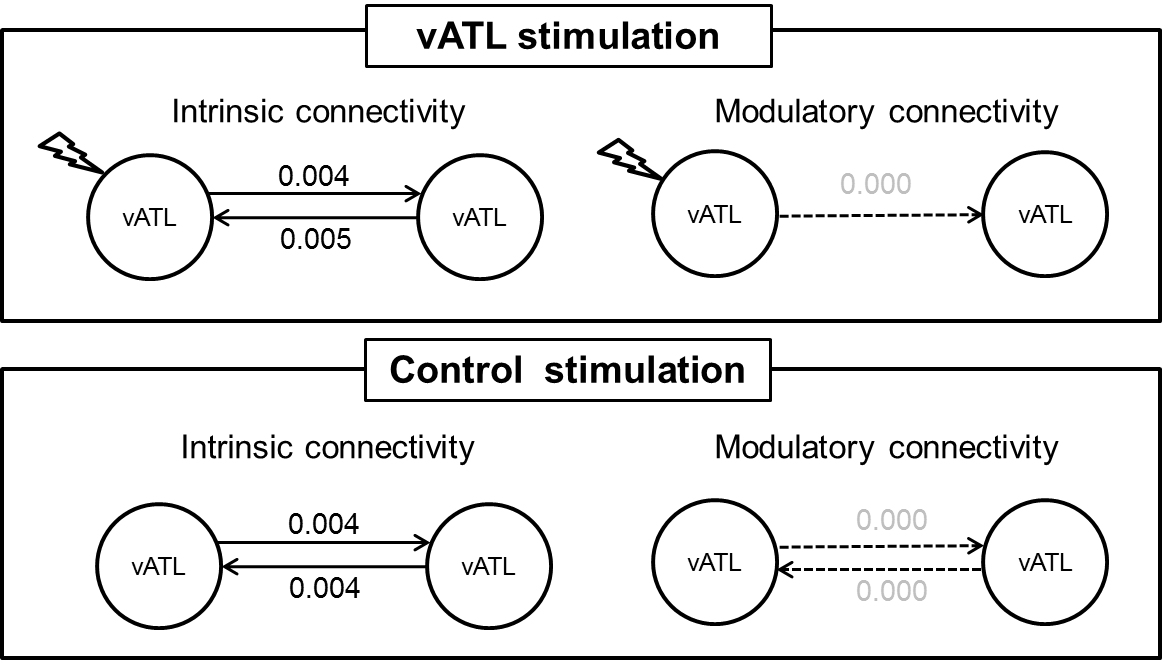
**

Supplementary Figure 5. Control task DCM results. Solid arrow indicates significant connectivity (p < 0.01 Bonferroni-corrected). Dotted arrow indicates non-significant connectivity.

**Supplementary Table 1. Whole brain analysis of synonym judgement > number judgement (P_FWE-corrected_ < 0.05).**

| Region | MNI coordinates | | | T | Z |
| --- | --- | --- | --- | --- | --- |
|  | x | y | z |  |  |
| vATL stimulation |  |  |  |  |  |
| Ventrolateral PFC | -57 | 15 | 21 | 10.94 | 6.43 |
|  | -30 | 30 | -12 | 7.32 | 5.48 |
|  | -33 | 33 | 3 | 10.98 | 6.44 |
|  | 36 | 30 | 3 | 7.51 | 5.24 |
| Ventral ATL | -39 | -15 | -27 | 6.59 | 5.25 |
|  | -36 | -15 | -30 | 6.41 | 5.19 |
| Posterior middle temporal gyrus | -57 | -36 | 6 | 11.65 | 6.58 |
|  | -54 | -39 | 9 | 11.06 | 6.45 |
| Premotor cortex | -45 | -3 | 51 | 6.43 | 5.2 |
| Supplementary motor area | -3 | 15 | 54 | 5.32 | 4.22 |
|  | 3 | 9 | 48 | 4.35 | 4.41 |
| Occipital cortex | -12 | -87 | -12 | 18.38 | 7.76 |
|  | 18 | -81 | -6 | 17.03 | 7.57 |
|  | 9 | -87 | -3 | 15.13 | 7.25 |
| Control stimulation |  |  |  |  |  |
| Ventrolateral PFC | -54 | 12 | 18 | 12.88 | 6.82 |
|  | -51 | 15 | 15 | 11.86 | 6.62 |
|  | 57 | 33 | 6 | 5.57 | 4.9 |
|  | 39 | 15 | 12 | 4.55 | 4.49 |
| Ventral ATL | -33 | -9 | -39 | 8.13 | 5.72 |
|  | -39 | -15 | -30 | 6.91 | 5.35 |
| Posterior middle temporal gyrus | -57 | -39 | 6 | 6.91 | 5.35 |
|  | -57 | -48 | 9 | 6.56 | 5.24 |
| Premotor cortex | -48 | -3 | 39 | 8.18 | 5.73 |
| Supplementary motor area | 0 | 6 | 57 | 7.89 | 5.38 |
|  | -6 | 12 | 45 | 7.55 | 5.29 |
|  | 12 | 18 | 33 | 5.24 | 4.59 |
| Occipital cortex | -12 | -90 | -9 | 18.71 | 7.81 |
|  | 12 | -87 | -3 | 18.05 | 7.72 |
|  | 9 | -87 | 9 | 14.93 | 7.13 |
|  | -21 | -87 | 18 | 13.09 | 6.85 |

**Supplementary Table 2. Parameter estimates of the winning model for the control stimulation**

| Parameter | Mean | SD | t | p |
| --- | --- | --- | --- | --- |
| Intrinsic connection |  |  |  |  |
| Left vATL → right vATL | 0.004 | 0.007 | 59.88 | < 0.001** |
| Right vATL → left vATL | 0.004 | 0.001 | 37.01 | < 0.001** |
| Left VC → left vATL | -0.001 | 0.006 | -2.42 | < 0.01** |
| Right VC → right vATL | -0.024 | 0.008 | -3.42 | < 0.01** |
| Modulation by cTBS |  |  |  |  |
| Left vATL → right vATL | -0.0001 | 0.01 | 1.17 | 0.535 |
| Right vATL → left vATL | 0.003 | 0.016 | 1.06 | 0.301 |
| Driving input |  |  |  |  |
| Left visual input | 0.023 | 0.006 | 7.842 | < 0.001** |
| Right visual input | 0.061 | 0.018 | 6.363 | < 0.001** |

** P < 0.01, two-tailed, Bonferroni-corrected.

**Supplementary Table 3. Parameter estimates of the winning model for the control task**

| TMS site | Parameter | Mean | SD | t | p |
| --- | --- | --- | --- | --- | --- |
| vATL stimulation | Intrinsic connection |  |  |  |  |
|  | Left vATL → right vATL | 0.004 | 0.001 | 452.790 | < 0.001** |
|  | Right vATL → left vATL | 0.005 | 0.000 | 14.760 | < 0.001** |
|  | Left VC → left vATL | 0.004 | 0.001 | 8.580 | < 0.001** |
|  | Right VC → right vATL | 0.009 | 0.009 | 6.500 | < 0.001** |
|  | Modulation by cTBS |  |  |  |  |
|  | Right vATL → left vATL | 0.000 | 0.000 | 1.640 | 0.116 |
|  | Driving input |  |  |  |  |
|  | cTBS over left vATL | 0.000 | 0.001 | 1.260 | 0.22 |
|  | Left visual input | 0.002 | 0.001 | 10.360 | < 0.001** |
|  | Right visual input | 0.003 | 0.001 | 13.970 | < 0.001** |
| Control stimulation | Intrinsic connection |  |  |  |  |
|  | Left vATL → right vATL | 0.004 | 0.001 | 197.870 | < 0.001** |
|  | Right vATL → left vATL | 0.004 | 0.000 | 226.170 | < 0.001** |
|  | Left VC → left vATL | 0.004 | 0.005 | 4.203 | < 0.001** |
|  | Right VC → right vATL | 0.009 | 0.009 | 5.228 | < 0.001** |
|  | Modulation by cTBS |  |  |  |  |
|  | Left vATL → right vATL | 0.000 | 0.000 | 1.874 | 0.074 |
|  | Right vATL → left vATL | 0.000 | 0.000 | 2.468 | 0.022 |
|  | Driving input |  |  |  |  |
|  | Left visual input | 0.002 | 0.001 | 7.970 | < 0.001** |
|  | Right visual input | 0.003 | 0.001 | 9.790 | < 0.001** |

** P < 0.01, two-tailed, Bonferroni-corrected.
